# Supplementary material for: Bioinspired 3D structures with programmable morphologies and motions
Source: Nat Commun. 2018 Sep 12;9:3705. doi: 10.1038/s41467-018-05569-8 (PMC6135848; doi:10.1038/s41467-018-05569-8)
Supplement: Supplementary file 1 — Supplementary Information [file 41467_2018_5569_MOESM1_ESM.pdf]

Supplementary Information

Bioinspired 3D structures with programmable morphologies and motions

Nojoomi et al.

Supplementary Note 1. Theoretical model

The theoretical model is based on the theory of differential geometry of surfaces[1,2] and the concept of target metrics (non-Euclidean plates)[3,4]. A differential growth (swelling or shrinking)-induced 3D shape (surface) adopts an isotropic embedding of a target metric[3-5]. The metric encodes the local equilibrium distances between points on the 3D shape (surface)[1-4]. Because bending ($E_{\mathrm{B}} \sim t_{\mathrm{h}}{}^{3}$, where t_{h} is the thickness of a sheet) is energetically less costly than stretching ($E_{\mathrm{S}} \sim t_{\mathrm{h}}$) in a thin sheet, the internal stresses developed by nonuniform in-plane growth are released by out-of-plane bending deformation ($E_{\mathrm{B}} < E_{\mathrm{S}}$)[3-5]. As the thickness of the sheet decreases, the shape converges to the embedding of the lowest bending energy[3-5].

We consider a 3D shape (suface) with a parameterization[1,2]

$$\mathbf{x}(u, v) = (x_1(u, v), x_2(u, v), x_3(u, v)), \tag{1}$$

where (u, v) are points on the 2D plane. The square of the element of arc length, or the distance between neighboring points, in the 3D surface is given by the first fundamental form (or the metric)

$$ds^2 = Edu^2 + 2Fdudv + Gdv^2, \tag{2}$$

where E, F, and G are the coefficients of the first fundamental form[1,2]. We assume that a spatially-controlled in-plane growth (swelling or shrinking) of a 2D plane in a coordinate system (u', v') can induce the formation of the 3D shape via out-of-plane deformation[3,4]. The square of the distance between points on the 2D plane before deformation is

$$dl^2 = \mathbf{g}du'dv', \tag{3}$$

where $\mathbf{g}$ is the metric (or first fundamental form) of the 2D plane before deformation[4]. The spatially controlled growth determines new equilibrium distances between points on the 2D

plane. The information of the new distances is contained in a new metric $\bar{\mathbf{g}}$. To form the 3D shape with the growth, the new metric of the 2D plane should be the same as that of the 3D shape:

$$ds^2 = \bar{\mathbf{g}} du' dv'. \tag{4}$$

We define the new metric $\bar{\mathbf{g}}$ as the target metric[3,4]. If the target metric (or the parameterization) is isothermal (or conformal), which is the case of our material systems ($E = G$ and $F = 0$), we can write the target metric with scale function λ

$$\bar{\mathbf{g}} = \lambda^2 \mathbf{g}. \tag{5}$$

We define $\boldsymbol{\Omega} = \bar{\mathbf{g}}$ as our target metric and λ^2 (or Ω) as our areal growth function[1,2]. $\boldsymbol{\Omega}$ contains all the information about how to encode a 2D plane with spatially controlled growth (swelling or shrinking) to form the target 3D shape. According to Gauss's *theorema egregium*, Gaussian curvature is then

$$K = -\Delta(\ln\lambda)/\lambda^2 = -\Delta(\ln\Omega)/(2\Omega), \tag{6}$$

where Δ is the Laplacian[1,2].

Supplementary Note 2. Theoretical model for axisymmetric 3D structures

We consider an axisymmetric 3D shape (surface of revolution) in a cylindrical coordinate system (ρ, φ, z), where the z axis is the axis of symmetry (axis of rotation) and $z = f(\rho)$. Then, we get the square of the element of arc length on the 3D shape from Supplementary Equation 2: $ds^2 = \left(1 + {f_\rho}^2\right) d\rho^2 + \rho^2 d\varphi^2$, where $f_\rho = df/d\rho$[1,2]. We assume that we can induce the 3D shape by encoding a 2D plane with Ω in a polar corodinate system (r, θ). Then, we get the following equation from Supplementary Equation 4:

$$\left(1 + f_\rho{}^2\right)d\rho^2 + \rho^2 d\varphi^2 = \Omega(r)(dr^2 + r^2 d\theta^2). \tag{7}$$

The left side of Supplementary Equation 7 represents the distance between two neighboring points on the 3D shape, whereas the right side represents the distance between neighboring points on the 2D plane after growth (swelling or shrinking). In other words, Supplementary Equation 7 describes how the spatially-controlled growth of the 2D plane (right side) induces the 3D shape (left side). Because the growth is axisymmetric, we assume that the angle between neighboring points on the 2D plane does not change during growth and thus obtain $d\varphi = d\theta$ and $\rho^2 d\varphi^2 = \Omega(r) r^2 d\theta^2$. Then, Supplementary Equation 7 gives

$$\rho^2 = \Omega(r) r^2, \tag{8}$$

$$\left(1 + f_\rho{}^2\right)d\rho^2 = \Omega(r) dr^2. \tag{9}$$

For a given axisymmetric 3D shape, we can thus determine the relationship of r and ρ and that of Ω and ρ. For a given Ω, we can predict the 3D shape that adopts Ω using Supplementary Equations 8 and 9.

Supplementary Note 3. Determination of the growth function (target metric) for a target 3D structure

We determine Ω for a spherical cap. A spherical cap with a radius of r_0 is given by

$$\rho^2 + (z - z_0)^2 = r_0{}^2. \tag{10}$$

where z_0 is a constant. We can then get ρ and Ω as a function of r using Supplementary Equations 8, 9, and 10

$$\rho = \frac{2r_0(r/R)}{1+(r/R)^2}, \tag{11}$$

$$\Omega = \frac{c}{(1+(r/R)^2)^2}, \tag{12}$$

where R and $c = 4(r_0/R)^2$ are constants. We can obtain local Gaussian curvature $K = 1/{r_0}^2 = 4/(cR^2)$ using Supplementary Equations 6 and 12. We used Supplementary Equation 12 to experimetally create a spherical cap at the shrunk state (Fig. 2a) and Supplementary Equation 10 to construct a theoretical 3D structure and K (Fig. 2d).

We next determine Ω for a cone structure. A cone with a vertex angle of 2β is given by

$$z - z_0 = \rho \cot \beta. \tag{13}$$

We then can get ρ and Ω as a function of r using Supplementary Equations 8, 9, and 13

$$\rho = \rho_0 \left(\frac{r}{R}\right)^{\alpha}, \tag{14}$$

where R is a constant and exponent $\alpha = \sin\beta$, and

$$\Omega = c \left(\frac{r}{\mathrm{R}}\right)^{2(\alpha-1)}, \tag{15}$$

where $c = (\rho_0/R)^2$. We can obtain $K = 0$ using Supplementary Equations 6 and 15. We used Supplementary Equation 15 to experimetally create a cone structure with a vertex angle of 2β at the shrunk state (Fig. 2c) and Supplementary Equation 13 to construct a theoretical 3D structure and K (Fig. 2f), respectively.

For a saddle shape, we used

$$\Omega = \frac{c}{(1-(r/R)^2)^2}. \tag{16}$$

We can then obtain $K = -4/(cR^2)$ using Supplementary Equations 6 and 16. We used Supplementary Equation 16 to experimentally create a saddle structure (Fig. 2b). We

constructed a theoretical 3D structure with constant negative K (Fig. 2e) by introducing two principle curvatures [$k_1 = -k_2 = 2/(\sqrt{c}R)$] into a flat surface using 3ds Max.

Supplementary Note 4. Prediction of a 3D shape from a growth function (target metric)

We demonstrate the prediction of 3D shapes using a growth function in the form

$$\Omega(r) = c\left[1 + \left(\frac{r}{R'}\right)^2\right]^{\alpha-1}, \quad (17)$$

where $R' = aR$ and a and α are constants (Fig. 2l and Supplemenatry Figure 6). The constant a is included to make Ω with different α have the same maximum and minimum values ($\Omega_{\max}$ and $\Omega_{\min}$) in $0 < r/R < 1$. We used the experimentally accessible maximum and minimum values of A_{35}/A_0 (Fig. 1b) as $\Omega_{\max}$ and $\Omega_{\min}$. We predicted 3D shapes using Supplementary Equations 8, 9, and 17 as a function of $\xi = r/R$

$$z(\xi) = \frac{{}_2F_1}{3(1+\alpha)\xi}\left[\frac{(1+\alpha)(a^2+\xi^2)}{a^2(-1+\alpha)}\right]^{\frac{3-\alpha}{2}}[2a^2 + (1+\alpha)\xi^2]$$
$$\sqrt{-\frac{1}{(a^2+\xi^2)^3}\left[a^2c^2(-1+\alpha)\xi^2\left(1+\frac{\xi^2}{a^2}\right)^{\alpha}(2a^2+(1+\alpha)\xi^2)\right]}, \quad (18)$$

where ${}_2F_1$ is a hypergeometric function ${}_2F_1 = \left[\frac{3}{2}, \frac{3-\alpha}{2}; \frac{5}{2}; -\frac{2a^2+(1+\alpha)\xi^2}{a^2(-1+\alpha)}\right]$. We constructed the theoretical 3D shapes and K as a function ρ, in which $\xi = r/R$ in Supplementary Equation 18 was converted to ρ using Supplementary Equation 8 (Figure 2l, Supplementary Figure 6). We used Mathematica (Wolfram) and MATLAB for the calculations. We calculated the theoretical values of the base angle γ

$$tan(\gamma) = \frac{(a^2+\xi^2)(1+\xi^2/a^2)^{(1-\alpha)/2}}{c(a^2+\alpha\xi^2)}\sqrt{-\frac{a^2c^2(-1+\alpha)\xi^2(1+\xi^2/a^2)^{\alpha}[2a^2+(1+\alpha)\xi^2]}{(a^2+\xi^2)^3}}. \quad (19)$$

We used Supplementary Equation 19 to plot the theoretical values of γ as a function of α and compared them with the experimentally obtained values in Fig. 2n.

Supplementary Note 5. Design and fabrication of stingray-inspired 3D structures

We designed and fabricated stingray-inspired 3D structures (Fig. 4m, n) based on the 3D morphology and swimming motions of stingrays in literature[6,7]. We first reconstructed and rendered the 3D morphology of a stingray using a computer-aided 3D modeling and rendering tool (3ds Max, Autodesk) as shown in Supplementary Figure 13a. We then calculated K and H^2 from the reconstructed 3D image using MATLAB (Fig. 4k, Supplementary Figure 13b). To fabricate the stingray-inspired 3D structure (Fig. 4m), we designed the modular components that mimic K and the morphologies of the body and the pectoral fins of the stingray model (Fig. 4k, l, Supplementary Figure 13). For the body structure with $K > 0$ (module 1), we designed Ω_{body} by transforming $\Omega(r) = c[1 + (r/R')^2]^{\alpha-1}$ with $\alpha = 0.8$ ($K > 0$; Fig. 2l, m, Supplementary Figure 6) to $\Omega(r, \theta) = \Omega(r/(a(\theta)R))$, where $a(\theta) = \sqrt{1 + (b^2 - 1)\sin^2\theta}$ with $b = 0.5$. This transformation changes the axisymmetric 3D structure ($K > 0$) into an elongated structure with an aspect ratio of 2 ($b = 0.5$). For the pectoral fins with $K < 0$ (module 2), we designed Ω_{fin} by transforming $\Omega(r) = c[1 + (r/R')^2]^{\alpha-1}$ with $\alpha = 1.5$ ($K < 0$) to $\Omega(r, \theta) = \Omega(r/(a(\theta)R))$, where $a(\theta) = \sqrt{1 + (b^2 - 1)\sin^2\theta}$ with $b = 0.75$. $\Omega(r) = c[1 + (r/R')^2]^{\alpha-1}$ with $\alpha > 1$ induces a 3D structure with $K < 0$, which is a negative analog to the structures with $K > 0$ shown in Fig. 2l and Supplementary Figure 6. To merge the pectoral fins with the body, we introduced linear linkers between Ω_{body} and Ω_{fin}, as described in Fig. 3c and Supplementary Figure 8. The structure for the body ($K > 0$) along with the linkers functions as transitional components, which control the direction of deformation of the pectoral fins ($K < 0$) and their

orientation with respect to the body and thus synchronize the motions of the left and right fins in response to temperature cycles. Without proper linkers, the modular components can be randomly oriented (Supplementary Figure 14). We designed and fabricated the stingray-inspired 3D structure in Fig. 4n as described above, using two modular components: (i) the body structure ($K > 0$) and (ii) the pectoral fin structures ($K < 0$). The motions of the ray-inspired structures (Figs. 4m, n, 6d) were controlled by modulating temperature cycles between 31.5 °C and 33.5 °C in a temperature-controlled water bath. For detailed observations of shape changes, we changed the temperature slowly.

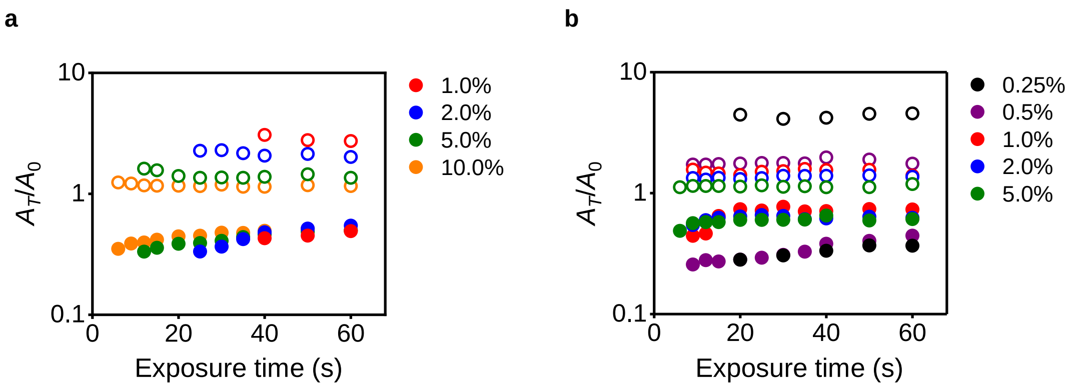

Supplementary Figure 1. Areal swelling and shrinking ratios of pNIPAm hydrogels crosslinked with single crosslinkers (BIS and PEGDA). (**a**) Areal swelling and shrinking ratios (A_T/A_0) of pNIPAm hydrogels crosslinked with BIS as a function of light exposure time. The hydrogels were prepared with BIS of 1.0 to 10.0 mol% of NIPAm in precursor solutions (as indicated in the legend). (**b**) Areal swelling and shrinking ratios (A_T/A_0) of pNIPAm hydrogels crosslinked with PEGDA as a function of light exposure time. The hydrogels were prepared with PEGDA of 0.25 to 5.0 mol% of NIPAm in precursor solutions (as indicated in the legend). The open and closed circles represent the swelling and shrinking ratios, respectively. The black, purple, red, blue, green, and orange circles represent A_T/A_0 of pNIPAm hydrogels crosslinked with crosslinkers (BIS and PEGDA) of 0.25, 0.5, 1.0, 2.0, 5.0, and 10.0 mol% of NIPAm in precursor solutions, respectively, as indicated in the legends. The results show that assuming the same kinetics of polymerization of NIPAm monomers with BIS and PEGDA, pNIPAm hydrogels crosslinked with long-chain crosslinkers (PEGDA) are formed at a lower monomer conversion (thus, lower network density at the gel point) than those crosslinked with short-chain crosslinkers (BIS).

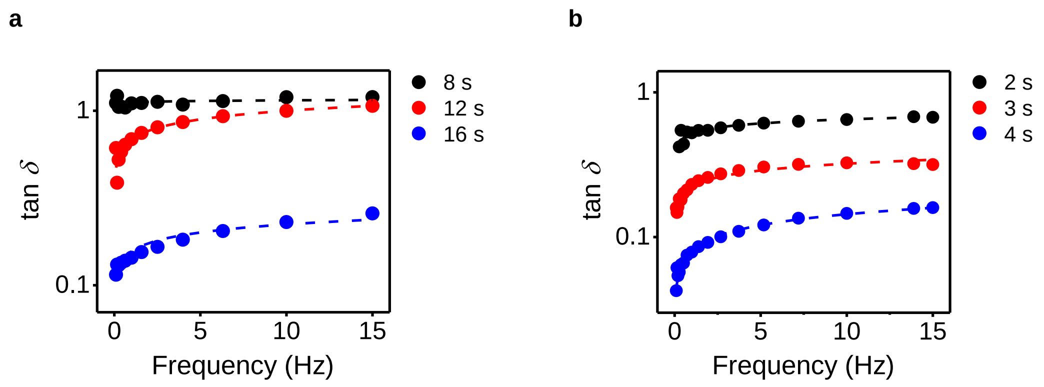

Supplementary Figure 2. Measurements of the gel points of pNIPAm hydrogels crosslinked with single crosslinkers (BIS and PEGDA). (**a**) tan δ of pNIPAm hydrogels crosslinked with BIS as a function of frequency. The black, red, and blue circles represent tan δ of pNIPAm hydrogels prepared by light exposure times of 8, 12, and 16 s, respectively. (**b**) tan δ of pNIPAm hydrogels crosslinked with PEGDA as a function of frequency. The black, red, and blue circles represent tan δ of pNIPAm hydrogels prepared by light exposure times of 2, 3, and 4 s, respectively. At the gel point, tan $\delta = G''/G'$ has a constant value over the frequency sweep, where G' and G'' are the shear storage modulus and shear loss modulus, respectively[8,9]. The measurements show that the prepolymer solutions with BIS and PEGDA form gels with light exposure times of around 8 s and less than 2 s, respectively.

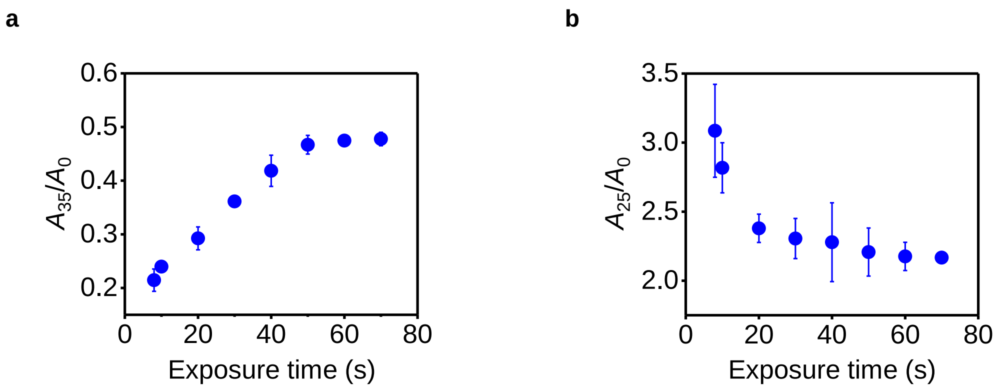

Supplementary Figure 3. Areal shrinking and swelling ratios of pNIPAm hydrogels crosslinked with both BIS and PEGDA. (**a**) Areal shrinking ratio (A_{35}/A_0) of pNIPAm hydrogels crosslinked with both BIS and PEGDA as a function of light exposure time. (**b**) Areal swelling ratio (A_{25}/A_0) of pNIPAm hydrogels crosslinked with both BIS and PEGDA as a function of light exposure time. The hydrogels were prepared with precursor solutions of NIPAm (0.2 g), BIS (0.5 mol% of NIPAm), PEGDA (0.25 mol% of NIPAm), and PBPO (0.3 mol% of NIPAm) in 1 mL aqueous solution (1:3 ratio of water and acetone by volume). The precursor solutions contain the same amount of BIS, PEGDA, and PBPO but 50 wt% of NIPAm in the precursor solutions used in Fig. 1b, c. Error bars: s.d. of three independent measurements.

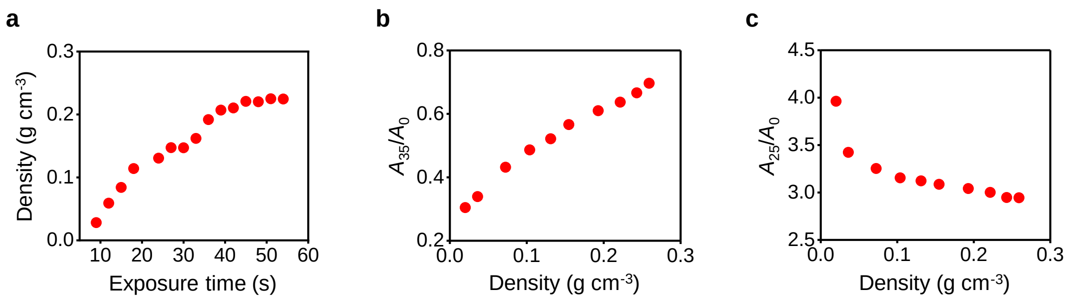

Supplementary Figure 4. Density of pNIPAm hydrogels. (**a**) The density of pNIPAm hydrogels crosslinked with BIS and PEGDA by different light exposure times was measured. The density of the pNIPAm hydrogels increases with light exposure time. The density was calculated using their dry mass and the volume of as-prepared hydrogels after washing with acetone and IPA. (**b**) Areal shrinking ratio (A_{35}/A_0) as a function of density. A_{35}/A_0 increases with the density of the hydrogels, showing that the degree of shrinking decreases with the density of the hydrogels. (**c**) Areal swelling ratio (A_{25}/A_0) as a function of density. A_{25}/A_0 decreases with the density of the hydrogels, showing that the degree of swelling decreases with the density of the hydrogels.

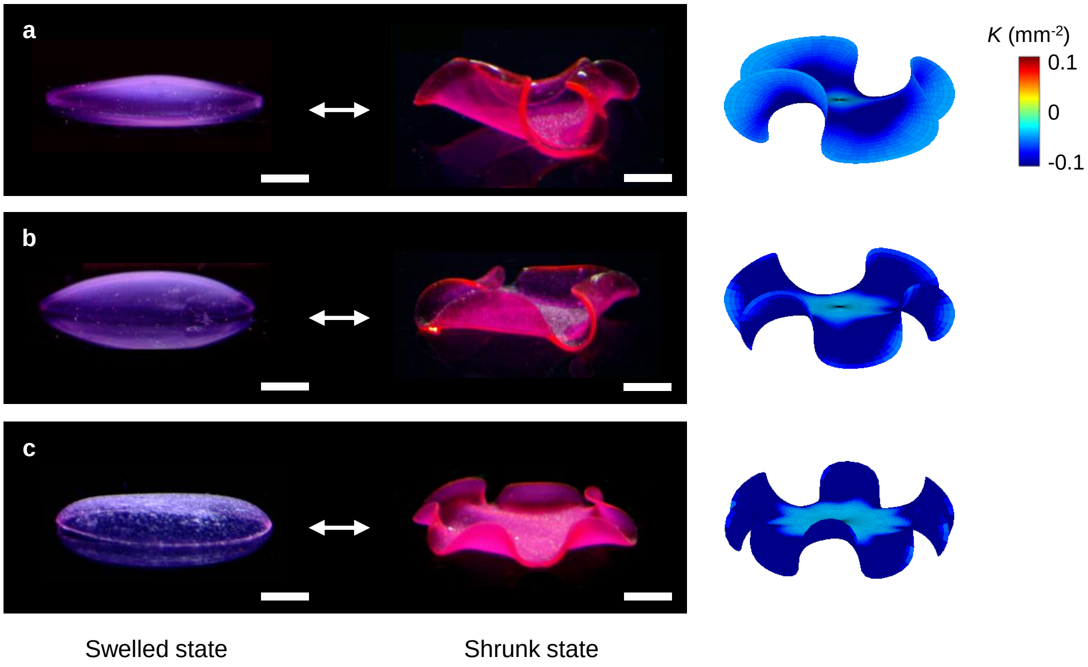

Supplementary Figure 5. Enneper's minimal surfaces with a different number of wrinkles. Experimentally created Enneper's minimal surfaces with $n' = 3$ (**a**), $n' = 4$ (**b**), and $n' = 6$ (**c**) at the shrunk state (middle). The images on the left side show the corresponding 3D structures at the swelled state. The images on the right side show the theoretical shapes of Enneper's minimal surfaces with $n' = 3$, $n' = 4$, and $n' = 6$. Scale bars, 5 mm (left); 2 mm (right).

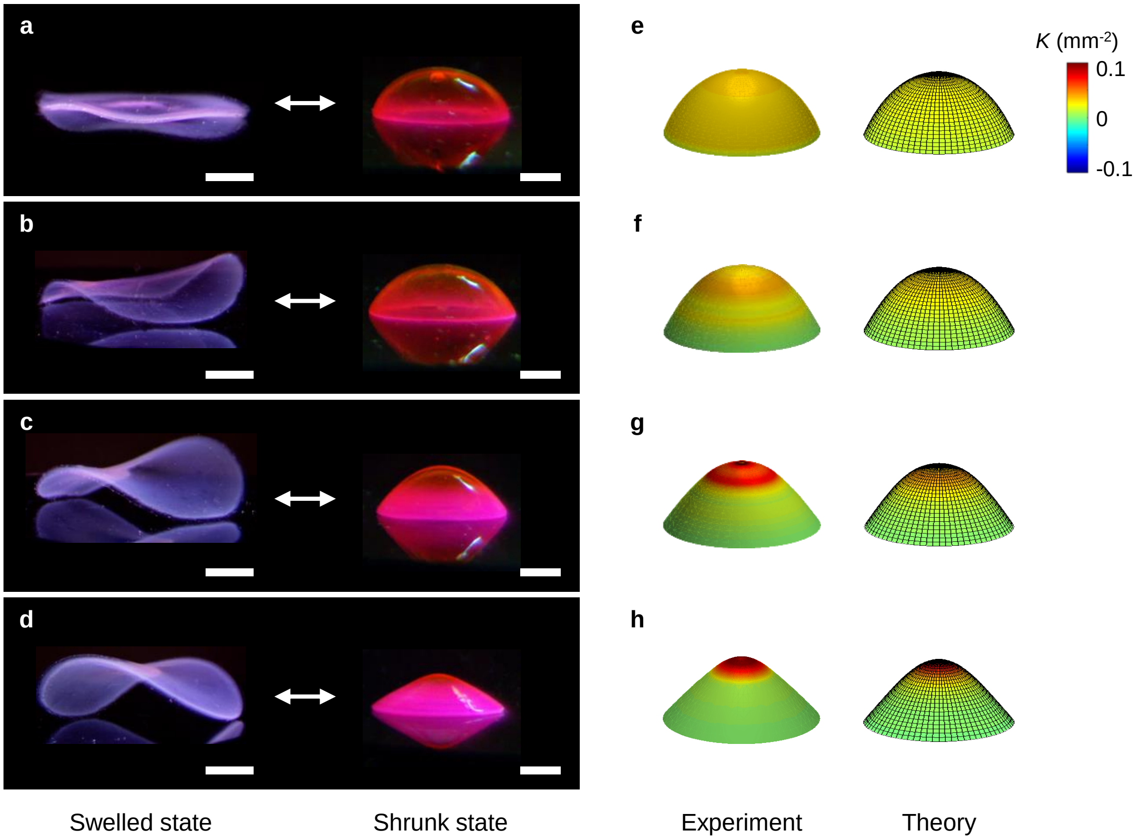

Supplementary Figure 6. Prediction and creation of 3D structures with a smooth gradient in Gaussian curvature *K*. (**a**–**d**) The 3D structures were created with $\Omega(r) = c[1 + (r/R')^2]^{\alpha-1}$, where $R' = aR$, with $\alpha = 0$ and $a = 0.94$ (**a**), $\alpha = 0.5$ and $a = 0.53$ (**b**), $\alpha = 0.75$ and $a = 0.22$ (**c**), which is also shown in Fig. 2l, and $\alpha = 0.9$ and $a = 0.022$ (**d**) (Fig. 2m). Scale bars, 5 mm (left); 2 mm (right). (**e**–**h**) Reconstructed 3D images with K of experimentally created (left) and theoretically predicted (right) 3D structures shown in **a**–**d**. The 3D images of the theoretically predicted 3D structures were constructed as described in Supplementary Note 4. The theoretical model predicts 3D shapes with a smooth gradient in K, which decreases from the maximum value to 0 with r, as shown in **e**– **h**. As compared in **e**–**h**, the experimental structures agree with the theoretical models.

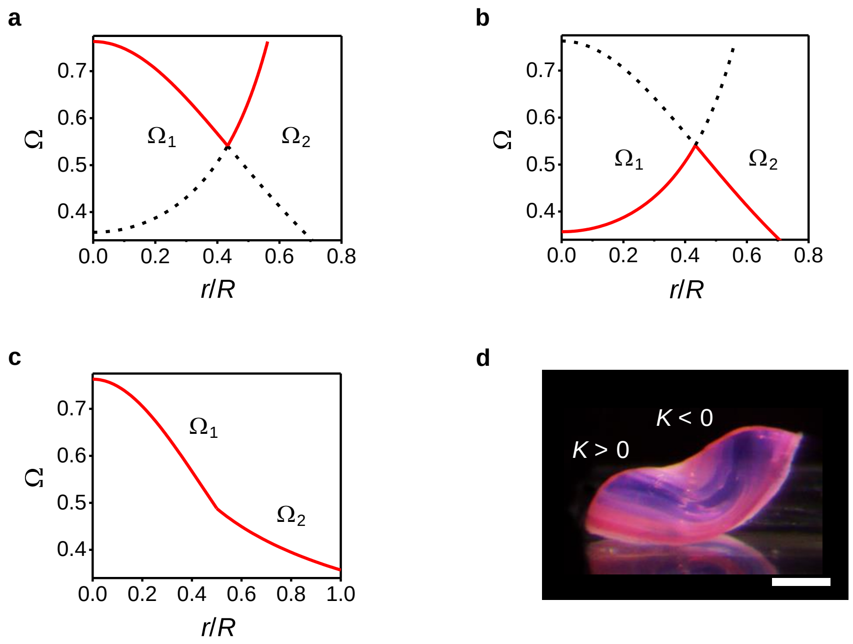

Supplementary Figure 7. Hybrid 3D structures with radially combined target metrics. (**a**) Ω (red) used to form the hybrid 3D structure shown in Fig. 3a. Ω radially combines Ω_1 (Ω for a spherical cap shown in Fig. 2a) at $0 < r/R < 0.43$ and Ω_2 (Ω for a saddle shape shown in Fig. 2b) at $0.43 < r/R < 0.56$. The black dashed lines show the projection of Ω_1 and Ω_2. (**b**) Ω that radially combines Ω_1 (Ω for a saddle shape shown in Fig. 2b) at $0 < r/R < 0.43$ and Ω_2 (Ω for a spherical cap shown in Fig. 2a) at $0.43 < r/R < 0.56$. The radially combined Ω were used to form the hybrid 3D structure shown in **d**. (**c**) Ω that radially combines Ω_1 (Ω for a spherical cap shown in Fig. 2a) at $0 < r/R < 0.5$ and Ω_2 (Ω for a cone shape with $\alpha = 0.775$ shown in Fig. 2c) at $0.5 < r/R < 1.0$. The radially combined Ω were used to form the hybrid 3D structure shown in Fig. 3b. (**d**) Hybrid 3D structure generated with Ω in **b**. The hybrid structure combines the saddle shape with $K < 0$ and the spherical cap shape with $K > 0$ in the center and outer regions, respectively. Scale bar, 2 mm.

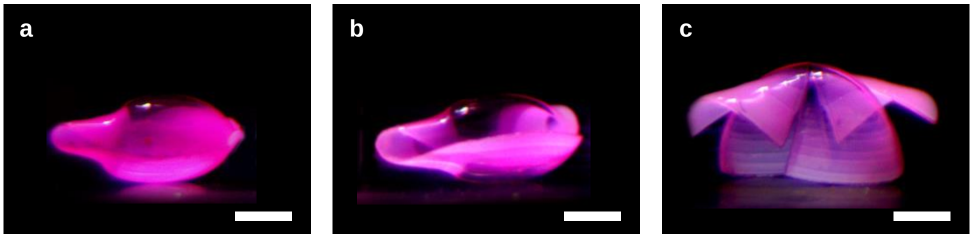

Supplementary Figure 8. Hybrid 3D structures with azimuthally combined target metrics. (**a**) Hybrid 3D structure created with Ω that azimuthally combines Ω_1 and Ω_2 shown in Fig. 3d without Ω_{L}. Ω_1 and Ω_2 are shown in Fig. 2m, which have with $\alpha = 0$ and $\alpha = 0.9$, respectively. The sharp discontinuities in Ω induce stress accumulation and thus shape distortion. (**b**) Hybrid 3D structure created with Ω that azimuthally combines Ω_1 and Ω_2 (Fig. 3d) with $\Omega_{\mathrm{L}} = (\Omega_1 - \Omega_2)(\theta/\Delta\theta) + \Omega_2$ with $\Delta\theta = 5°$ at the interfaces. Ω that combines Ω_1 and Ω_2 using Ω_{L} with $\Delta\theta = 5°$ induces shape distortion, because of sharp changes at the interfaces. (**c**) Hybrid 3D structure created with Ω that azimuthally combines Ω_1 and Ω_2 shown in Fig. 3d without Ω_{L} but with space $\Delta\theta = 5°$ at the interfaces of Ω_1 and Ω_2. The hybrid structure shows the key signatures of the structures induced by Ω_1 and Ω_2 along the θ direction (Supplementary Figure 6a, d). Ω_1 yields a spherical cap-like shape (Supplementary Figure 6a), whereas Ω_2 yields a shape that combines a spherical cap-like shape in the center and a cone-like shape with a large vertex angle in the edge (Supplementary Figure 6d). Scale bars, 2 mm.

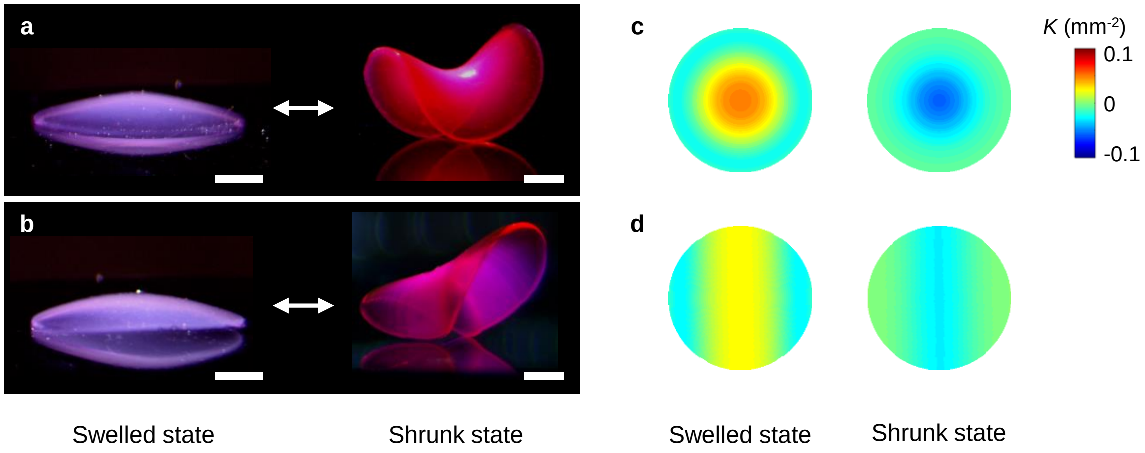

Supplementary Figure 9. 3D structures with continuously varying morphologies along the θ direction. (**a**, **b**) 3D structures created with $\Omega(r, \theta)$ in Fig. 3i with $L = 0$ (**a**) and $L = 1$ (**b**). The structures on the left and right sides are at the swelled and shrunk states, respectively. Scale bars, 5 mm (left); 2 mm (right). (**c**, **d**) Corresponding Gaussian curvature K maps of the structures with $L = 0$ (**c**) and $L = 1$ (**d**) at the swelled and shrunk states. Gaussian curvature K is calculated from Ω in Fig. 3i.

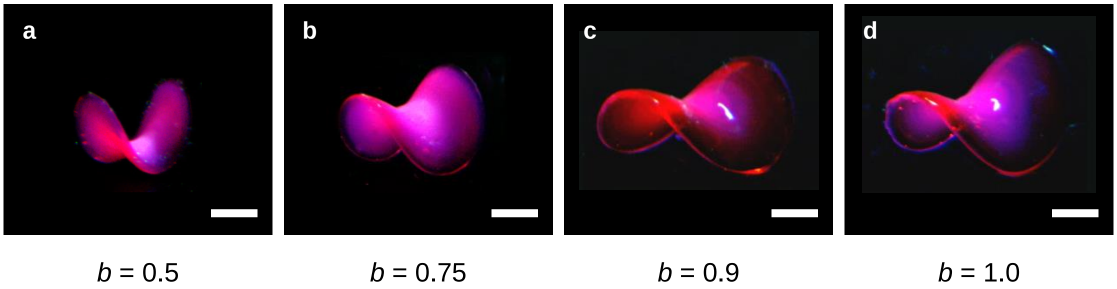

Supplementary Figure 10. Elongated elliptical saddle structures. The elongated elliptical saddle structures were created by transforming axisymmetric Ω for an saddle shape into a nonaxisymmetric form $\Omega(r, \theta) = c\Omega(r/(a(\theta)R))$, where $a(\theta) = \sqrt{1 + (b^2 - 1)\sin^2\theta}$. The major and minor axes of the ellipse are R and bR $(0 < b < 1)$ or bR and R $(b > 1)$, respectively. The elongated elliptical saddle structures were formed with $b = 0.5$ (**a**), $b = 0.75$ (**b**), $b = 0.9$ (**c**), and $b = 1.0$ (**d**), respectively. The structure with $b = 0.5$ in **a** is also shown in Fig. 3j. Scale bars, 2 mm.

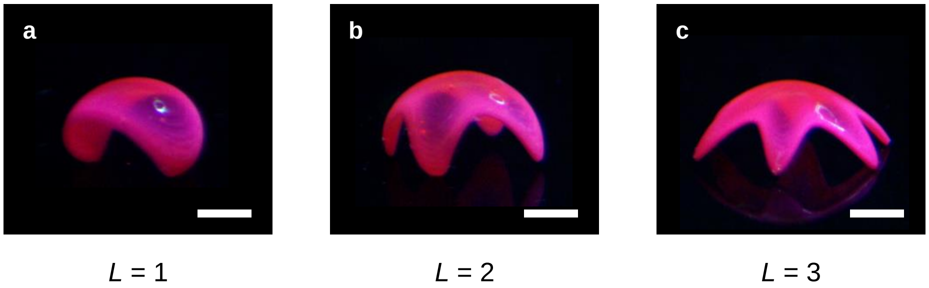

Supplementary Figure 11. Spherical caps with a targeted number of legs (nodes). The spherical caps with a targeted number of legs (nodes) were created by transforming axisymmetric $\Omega(r)$ for a spherical cap into a nonaxisymmetric form $\Omega(r,\theta) = c\Omega(r/(a(\theta)R))$, where $a(\theta) = \sqrt{1 + (b^2 - 1)\sin^2 L\theta}$. The transformed $\Omega(r, \theta)$ has the period of π/L along the θ direction and thus induces a 3D structure with $2L$ nodes (legs). b defines the ratio of the inner diameter to the outer diameter of the structure (and thus the length of the legs). The spherical caps with 2 (**a**), 4 (**b**), and 6 (**c**) legs were formed using $\Omega(r,\theta)$ with $b = 0.5$ and $L = 1$, 2, and 3, respectively. Scale bars, 2 mm.

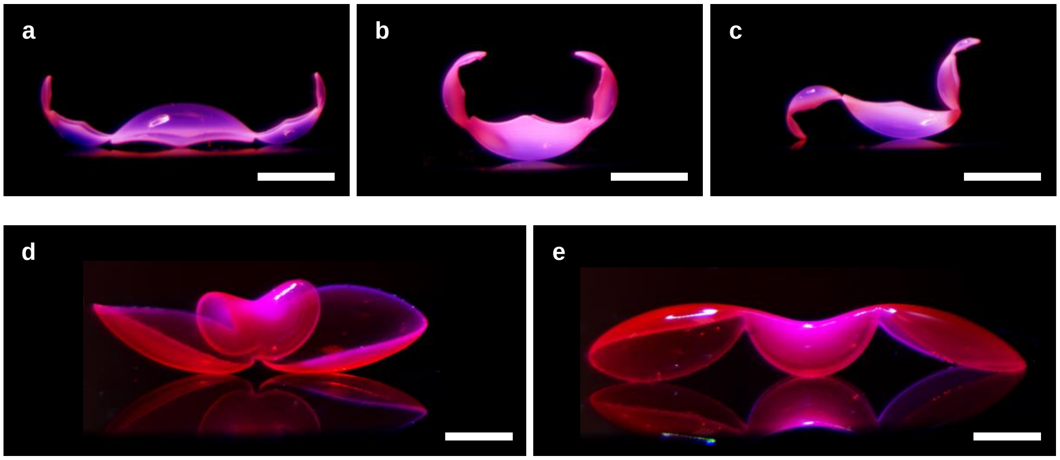

Supplementary Figure 12. Multimodular 3D structures with the same target metric but different conformations. (**a**–**c**) Multimodular structures that consist of a modular component with $K > 0$ (spherical cap with $R = 10$ mm in Supplementary Equation 12 in Supplementary Note 3) in the center and two smaller components with $K > 0$ (spherical caps with $R = 5$ and 2.5 mm in Supplementary Equation 12 in Supplementary Note 3) on the left and right sides. The three structures in **a**, **b**, and **c** were formed with the same growth function but have different conformations, as the modules can randomly select a direction of deformation (upward or downward) with respect to neighboring modules. Scale bars, 2 mm. (**d**, **e**) Multimodular structures that consist of a module with $K < 0$ in the center and two modules with $K > 0$ on the left and right sides. The two structures shown in **d** and **e** were formed with the same growth function but have different conformations, as the modules can randomly adopt an orientation with respect to neighboring modules. Scale bars, 4 mm.

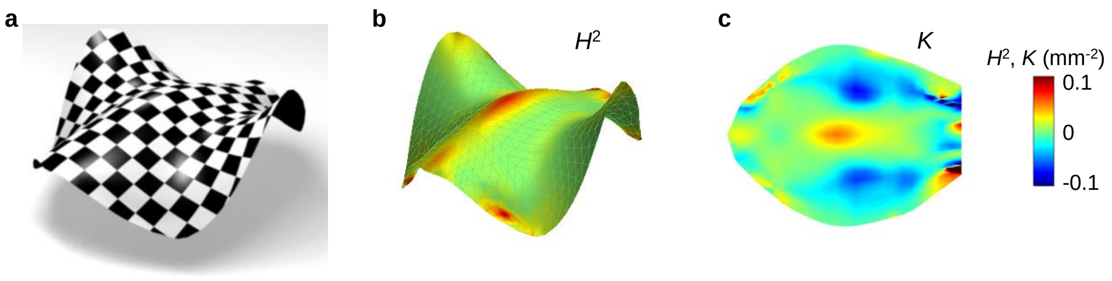

Supplementary Figure 13. Design of stingray-inspired 3D structures. a, Reconstructed 3D image and rendering of a stingray. The 3D image was reconstructed based on the 3D morphology of stingrays in literature[6,7]. **b**, Reconstructed 3D image of the stingray model with squared mean curvature H^2. **c**, Top-view of the reconstructed 3D image of the stingray model with K (Fig. 4k).

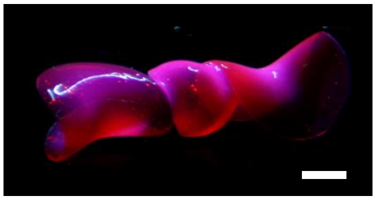

Supplementary Figure 14. Stingray-inspired 3D structure without linkers. The stingray-inspired 3D structure was constructed with the same modules for the body and the pectoral fins used in the structure in Fig. 4m but without linkers. Although they maintain the designed shape ($K < 0$), the pectoral fin structures are randomly oriented with respect to the body without linkers. Scale bar, 2 mm.

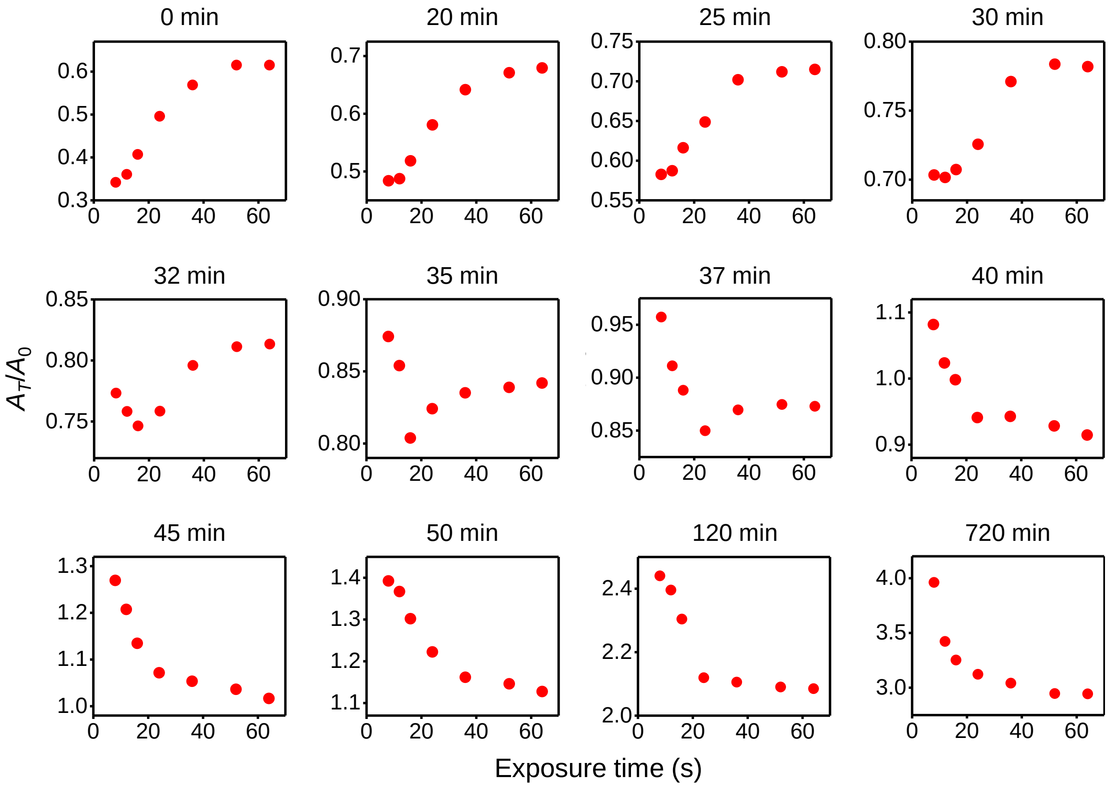

Supplementary Figure 15. Time-dependent areal swelling and shrinking ratios as a function of light exposure time at different times during cooling. The areal swelling and shrinking ratios as a function of light exposure time t_{ex} at different times t during cooling were constructed using Fig. 5b. $A_T/A_0(t_{\mathrm{ex}})$ changes from an increasing function of t_{ex} (shrunk state) to a decreasing function of t_{ex} (swelled state) with time. The transition of $A_T/A_0(t_{\mathrm{ex}})$ reflects how the spherical cap in Fig. 5a transforms from a shape with $K > 0$ at the shrunk state to a shape $K < 0$ at the swelled state.

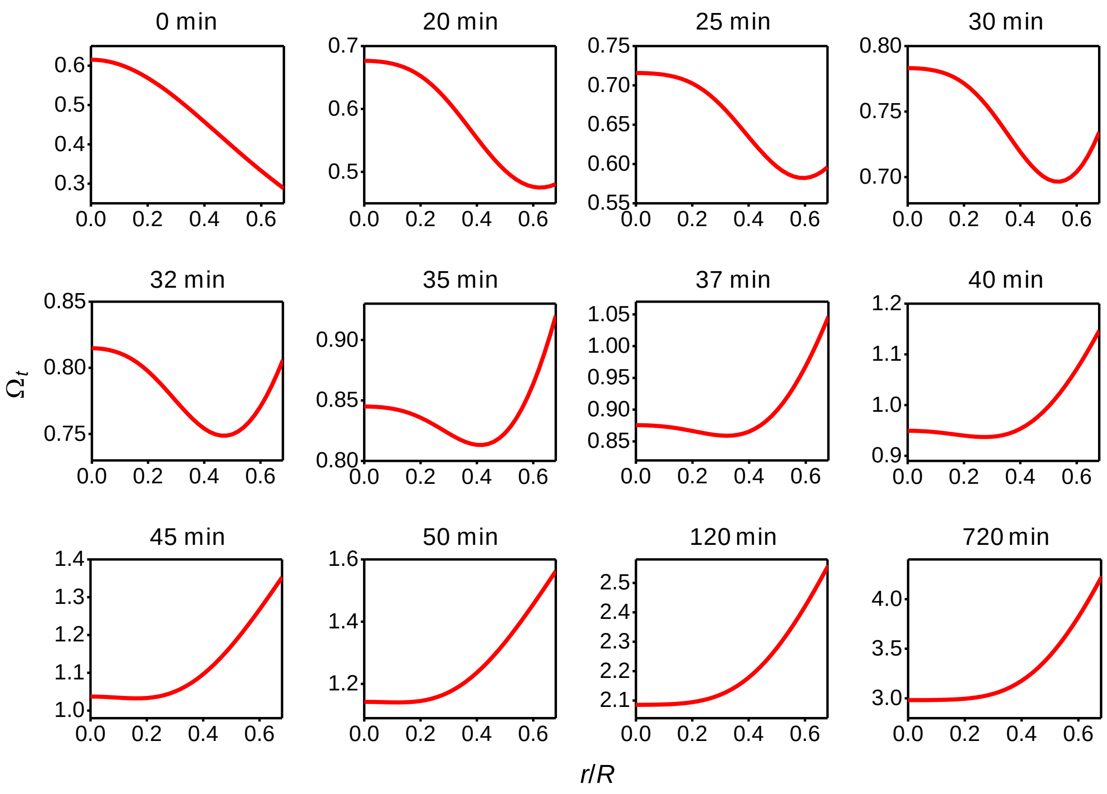

Supplementary Figure 16. Dynamic growth functions (target metrics) for a spherical cap structure at different times during cooling. The dynamic growth functions (or target metrics) Ω_t of the spherical cap at different times during cooling (Fig. 5a) were constructed from Ω for a spherical cap structure (Fig. 2g) using the dynamic calibration curves (Supplementary Figure 15). Ω_t changes from a decreasing function of r/R to an increasing function, reflecting the transformation of the spherical cap shape ($K > 0$) at the shrunk state to the saddle-like shape ($K < 0$) at the swelled state.

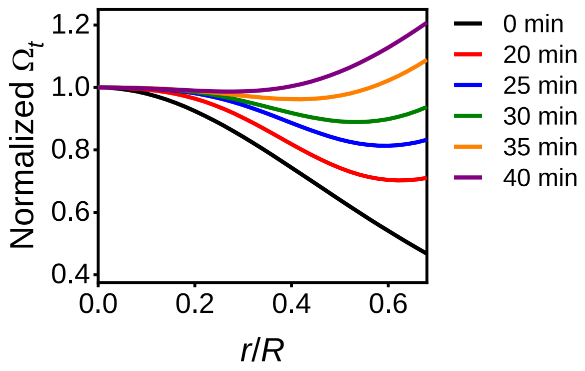

Supplementary Figure 17. Normalized dynamic Ω_t of the spherical cap structure at $t = 0$ to 40 min. Ω_t shown in Fig. 5d were normalized with respect to the value of Ω_t at $r/R = 0$.

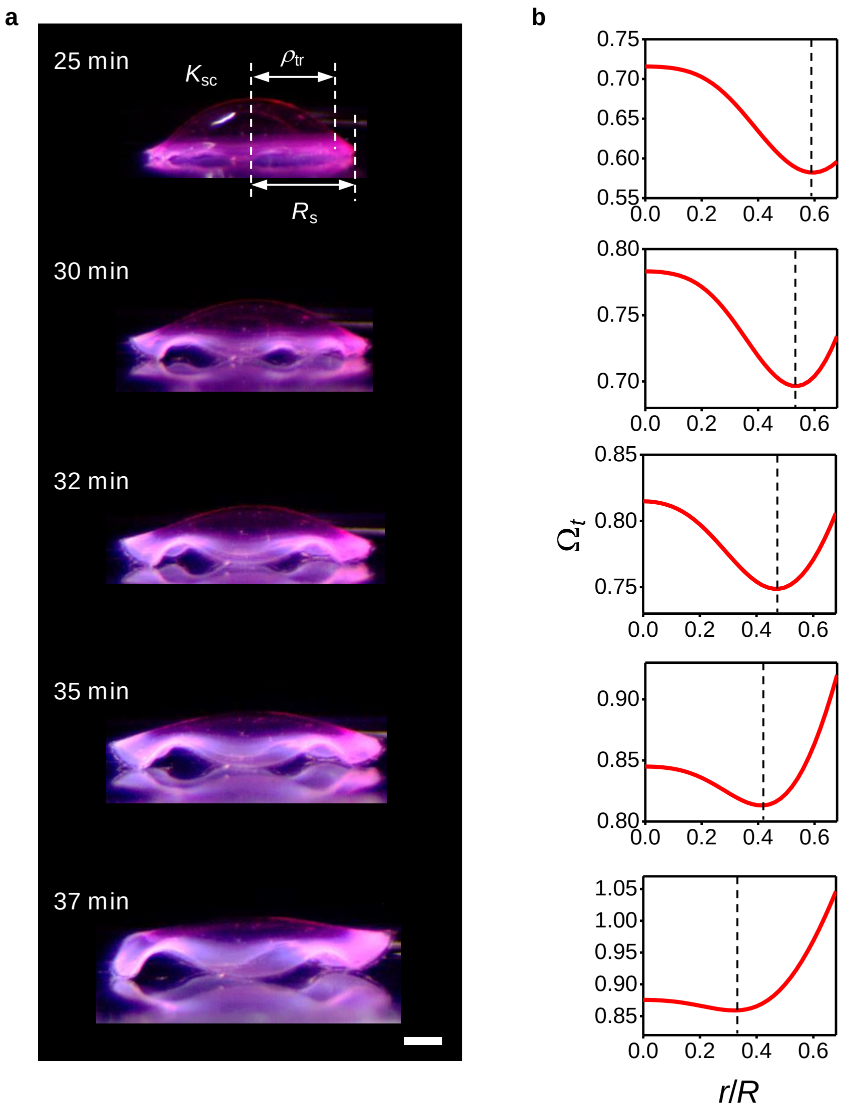

Supplementary Figure 18. Dynamic shapes of the spherical cap structure. **a**, Dynamic shapes of the spherical cap structure shown in Fig. 5a at 25, 30, 32, 35, and 37 min. The location of the shape transition between the spherical cap-like shape ($K > 0$) and the wrinkles ($K < 0$) $(\rho/R_s)_{tr}$ was obtained by measuring ρ_{tr} and R_s as shown in the structure at 25 min: $(\rho/R_s)_{tr} = \rho_{tr}/R_s$. Scale bar, 2 mm. **b**, Ω_t for the spherical cap at 25, 30, 32, 35, and 37 min. The location of the minimum $(r/R)_{min}$, indicated by the dashed lines in the graphs, shifts from the edge toward the center with time.

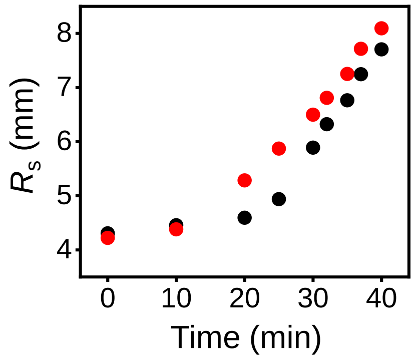

Supplementary Figure 19. Radius of the spherical cap structure as a function of time. The radius R_s of the dynamic shapes of the spherical cap structure shown in Fig 5a was measured as a function time (black circles) as shown in Supplementary Figure 18a. The theoretically calculated R_s (red circles) was obtained from Ω_t (Fig. 5d, e, Supplementary Figure 16) using Supplementary Equation 8 in Supplementary Note 2.

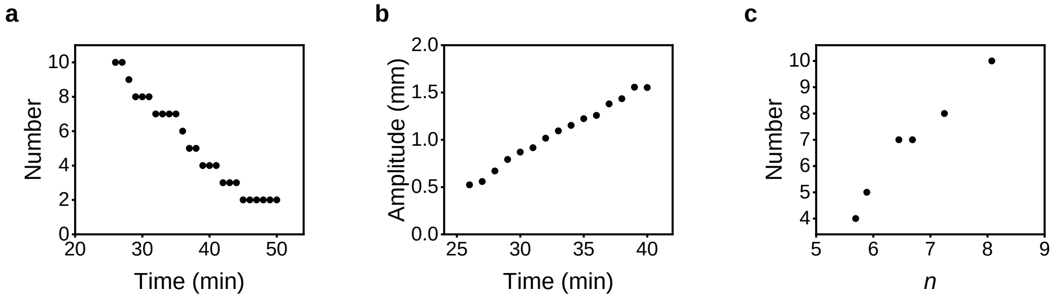

Supplementary Figure 20. Experimentally measured number and amplitude of the wrinkles in the dynamic shapes of the spherical cap structure. a, Experimentally measured number of the wrinkles of the spherical cap structure shown in Fig. 5a as a function of time. **b**, Experimentally measured maximum amplitude of the wrinkles of the structure as a function of time. **c**, Experimentally measured number of the wrinkles shown in **a** as a function of n. The values of n were obtained by fitting $\Omega = c/[1+(r/(aR))^2]^2 + [1+(r/R)^n]^2 - 1$ to Ω_t (shown in Fig. 5d, e and Supplementary Figure 16), where the first and second terms in Ω represent the spherical cap-like shape (a functional form of spherical caps in Fig. 2g) and the wrinkles (a functional form of Enneper's surfaces in Fig. 2k), respectively, and c, a, and n are constants. As observed in Enneper's surfaces (Fig. 2i–k, Supplementary Figure 5), the number of wrinkles in the structure increases with n.

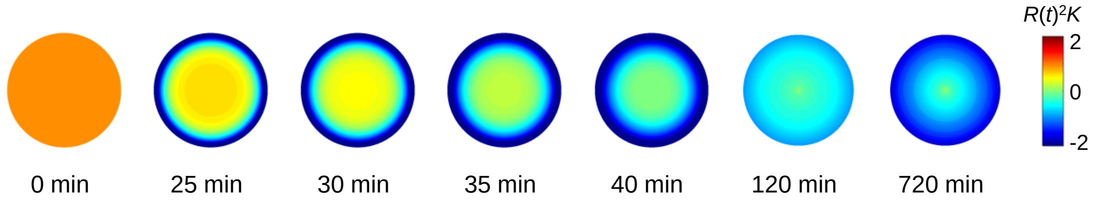

Supplementary Figure 21. Maps of normalized Gaussian curvature of the spherical cap structure at different times during cooling. The maps of normalized Gaussian curvature $R^2(t)K$ of the spherical cap structure at different times (Fig. 5a) were constructed from Ω_t shown in Supplementary Figure 16. The K maps reflect the experimentally observed shape transformations shown in Fig. 5a as described in the main text. $R(t)$ is the time-dependent R in the coordinate of 3D structures at the swelled and shrunk states (ρ, φ, z) calculated by Supplementary Equation 8 in Supplementary Note 2: $R(t)^2 = \Omega_t(R)R^2$.

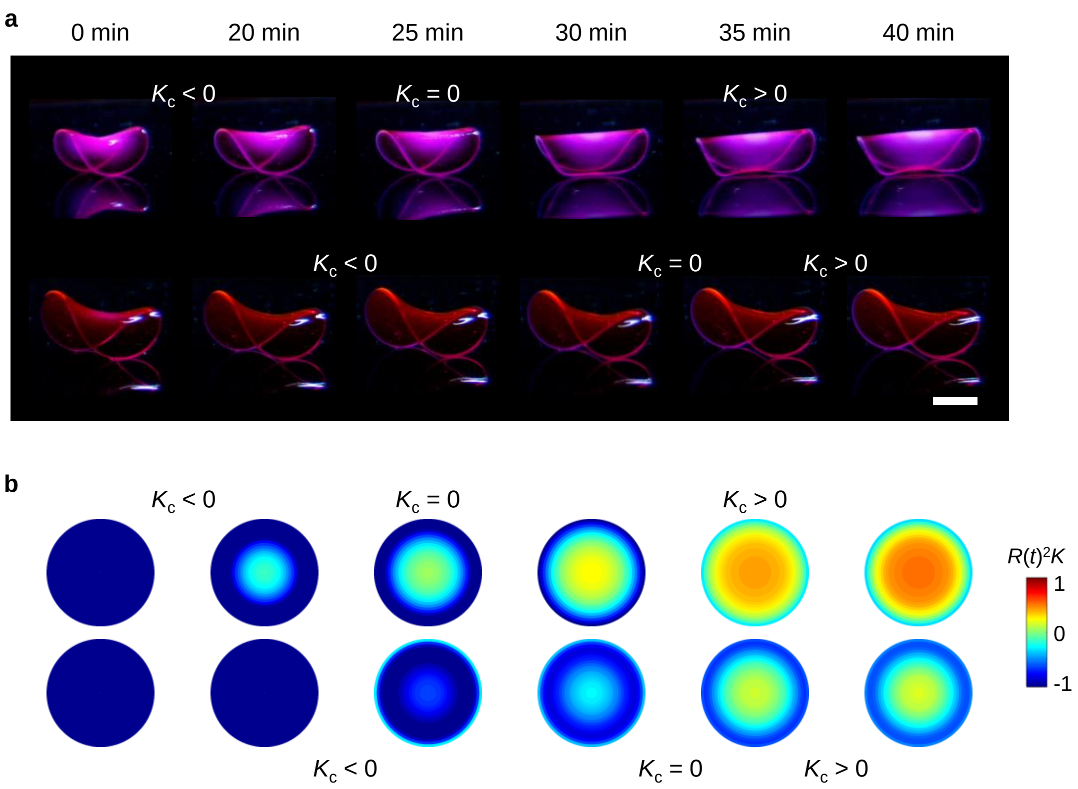

Supplementary Figure 22. Saddle structures with different speeds of shape transformation. **a**, Dynamic shape evolution of saddle structures ($K < 0$) with high and low speeds of shape transformation during cooling. The top (fast) and bottom (slow) structures were created with the growth functions in the low (red line) and high (blue line) ranges in Fig. 6c, respectively. The two structures have the same shape but different sizes because of the use of Ω in different ranges. As designed, the top structure transforms its shape faster than the bottom structure. For example, the top structure transforms from a shape with $K_c < 0$ to a shape with $K_c > 0$ around 25 min, whereas the bottom structure at around 30 to 35 min. K_c is Gaussian curvature in the center of the structures. Scale bar, 2 mm. **b**, Maps of normalized Gaussian curvature $R^2(t)K$ of the structures in **a**. The dynamic K maps were constructed using Ω_t for the saddle structures.

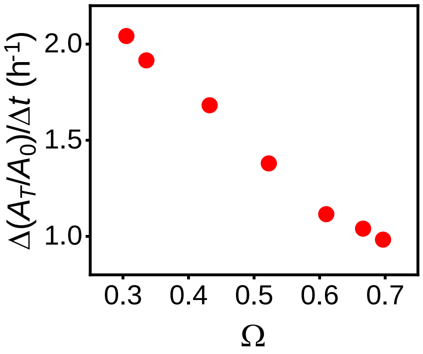

Supplementary Figure 23. Areal swelling rates (speeds of shape change) as a function of Ω.

The areal swelling rates $\Delta(A_T/A_0)/\Delta t$ were calculated using the swelling rates of the hydrogels as a function of t_{ex} (Fig. 1d) and the static calibration curve at the shrunk state (Fig. 1b). As the swelling rate decreases with a value of Ω, we can create structures with different speeds of shape change by programming the structures with different ranges of Ω (as shown in Fig. 6a–c and Supplementary Figure 22).

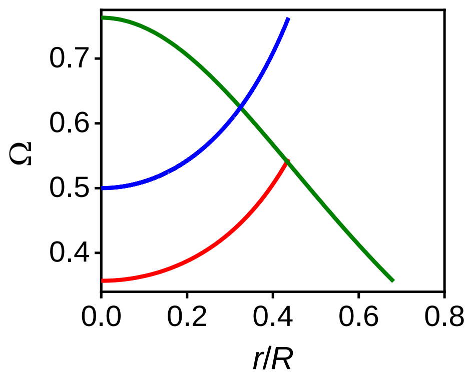

Supplementary Figure 24. Ω used to fabricate a ray-inspired structure with programmed sequential motions. Ω shown in green, red, and blue lines were used for the modules for the body, the front wings, and the rear wings of the structure shown in Fig. 6d, respectively.

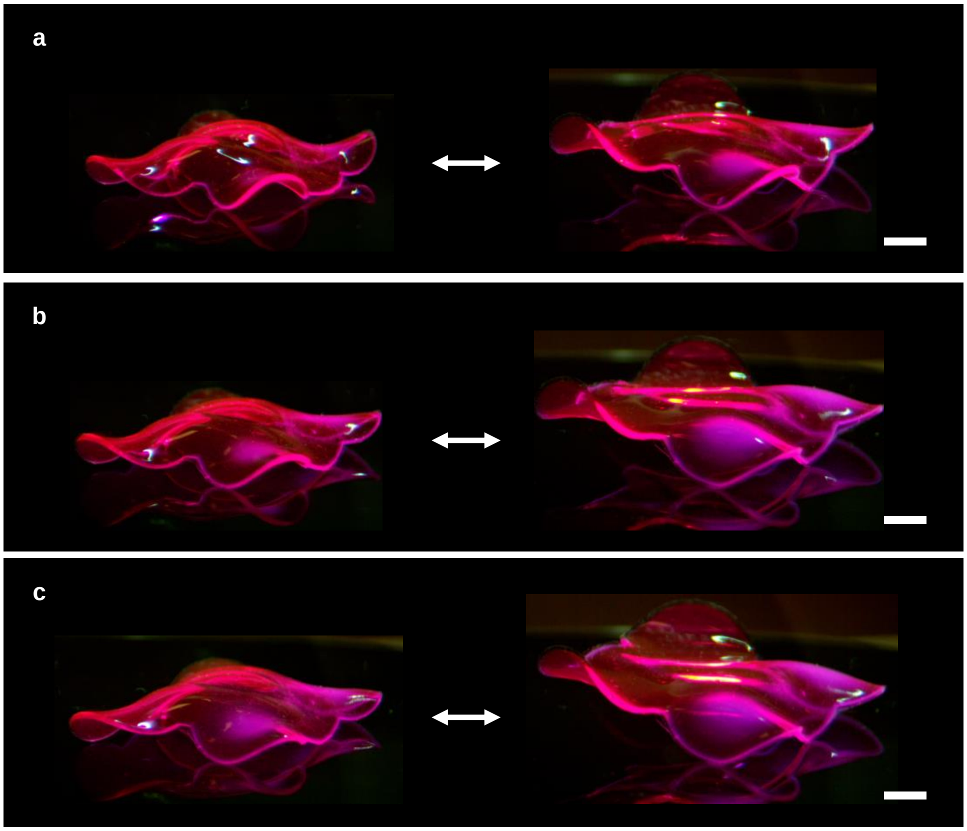

Supplementary Figure 25. Control of the motions of the ray-inspired structure by modulating temperature cycles. The motions of the ray-inspired structure can be controlled by modulating temperature cycles. The figures show 3 continuous cycles of the motions with 20 (**a**), 25 (**b**), and 30 (**c**) minute cooling times. By controlling the temperature cycle (e.g., cooling and heating times), the amplitude and frequency of the programmed motions can be controlled. The amplitude of the motions increases with increasing cooling times. The rear wings show a rapid snapping motion. This behavior is attributed to the transformation of stored elastic energy, resulted from the interactions of the rear wings and the surface, into the kinetic energy of the motions (Supplementary Movie 5)[10,11]. Scale bars, 2 mm.

Supplementary References

1. O'Neill, B. *Elementary differential geometry*. (Academic Press, New York, 1997).
2. do Carmo, M. P. *Differential geometry of curves and surfaces*. (Dover Publications, New York, 2016).
3. Klein, Y., Efrati, E. & Sharon, E. Shaping of elastic sheets by prescription of non-Euclidean metrics. *Science* **315**, 1116-1120 (2007).
4. Sharon, E. & Efrati, E. The mechanics of non-Euclidean plates. *Soft Matter* **6**, 5693-5704 (2010).
5. Kim, J., Hanna, J. A., Byun, M., Santangelo, C. D. & Hayward, R. C. Designing responsive buckled surfaces by halftone gel lithography. *Science* **335**, 1201-1205 (2012).
6. Blevins, E. L. & Lauder, G. V. Rajiform locomotion: Three-dimensional kinematics of the pectoral fin surface during swimming by freshwater stingray *Potamotrygon orbignyi*. *J. Exp. Biol.* **215**, 3231-3241 (2012).
7. Park, S.-J. *et al.* Phototactic guidance of a tissue-engineered soft-robotic ray. *Science* **353**, 158-162 (2016).
8. Winter, H. H. & Chambon, F. Analysis of linear viscoelasticity of a crosslinking polymer at the gel point. *J. Rheol.* **30**, 367-382 (1986).
9. Rodd, A. B., Cooper-White, J., Dunstan, D. E. & Boger, D. V. Gel point studies for chemically modified biopolymer networks using small amplitude oscillatory rheometry. *Polymer* **42**, 185-198 (2001).
10. Forterre, Y., Skotheim, J. M., Dumais, J. & Mahadevan, L. How the Venus flytrap snaps. *Nature* **433**, 421-425 (2005).

11. Fratzl, P. & Barth, F. G. Biomaterial systems for mechanosensing and actuation. *Nature* **462**, 442-448 (2009).
