# Supplementary material for: Bioinspired 3D structures with programmable morphologies and motions
Source: Nat Commun. 2018 Sep 12;9:3705. doi: 10.1038/s41467-018-05569-8 (PMC6135848; doi:10.1038/s41467-018-05569-8)
Supplement: Supplementary file 3 — Description of Additional Supplementary Files [file 41467_2018_5569_MOESM3_ESM.pdf]

Description of Additional Supplementary Files

File Name: Supplementary Movie 1
Description: Shape evolution of an Enneper's minimal surface. This movie shows the shape evolution of an Enneper's minimal surface with $n' = 6$ shown in Fig. 2j from the shrunk state to the swelled state. The movie is shown 300x faster than real time.

File Name: Supplementary Movie 2
Description: Shape evolution of a 3D structure with alternating $K > 0$ and $K < 0$. This movie shows the shape evolution of the 3D structure with alternating $K > 0$ and $K < 0$ along the θ direction shown in Fig. 3e. The structure reversibly transforms between the shapes at the shrunk state and the swelled state in response to temperature cycles. The movie is shown 300x faster than real time.

File Name: Supplementary Movie 3
Description: Motions of a stingray-inspired structure. This movie shows the oscillatory flapping motions of the stingray-inspired structure shown in Fig. 4m in response to temperature cycles. The movie is shown 600x faster than real time.

File Name: Supplementary Movie 4
Description: Motions of a stingray-inspired structure. This movie shows the oscillatory flapping motions of the stingray-inspired structure shown in Fig. 4n in response to temperature cycles. The movie is shown 600x faster than real time.

File Name: Supplementary Movie 5
Description: Motions of a ray-inspired 3D structure with programmed sequential motions. This movie shows three continuous cycles of the oscillatory motions of the ray-inspired 3D structure shown in Fig. 6d in response to temperature cycles with different cooling times (20, 25, and 30 min). The movie is shown 600x faster than real time.
